# Nurturing transformative local structures of multisectoral collaboration for primary health care: qualitative insights from select states in India

**DOI:** 10.1186/s12913-024-11002-2

**Published:** 2024-05-16

**Authors:** Shalini Singh, Emily Miller, Svea Closser

**Affiliations:** 1 Department of International Health, Johns Hopkins Bloomberg School of Public Health (JHSPH), Baltimore, USA; 2 Johns Hopkins India Private Limited, New Delhi, India

**Keywords:** Multisectoral collaboration, Intersectoral collaboration, Primary Healthcare, India, decentralization, local governments

## Abstract

**Background:**

Multisectoral collaboration is essential for advancing primary health care (PHC). In low- and middle-income countries (LMICs), limited institutional capacities, governance issues, and inadequate stakeholder engagement impede multisectoral collaboration. India faces similar challenges, especially at the meso-level (districts and subdistricts). Owing to its dependence on context, and insufficient evidence, understanding “How” to improve multisectoral collaboration remains challenging. This study aims to elicit specific recommendations to strengthen meso-level stewardship in India for multisectoral collaboration. The findings from this study may offer lessons for other LMICs.

**Methods:**

Using purposive, maximum variation sampling, the study team conducted semi-structured interviews with 20 diverse participants, including policymakers, implementers, development agency representatives, and academics experienced in multisectoral initiatives. The interviews delved into participants’ experiences, the current situation, enablers, and recommendations for enhancing stakeholder engagement and capacities at the meso-level for multisectoral collaboration.

**Results:**

Context and power are critical elements to consider in fostering effective collaboration. Multisectoral collaboration was particularly successful in three distinct governance contexts: the social-democratic context as in Kerala, the social governance context in Chhattisgarh, and the public health governance context in Tamil Nadu. Adequate health system input and timely guidance instil confidence among local implementers to collaborate. While power plays a role through local leadership’s influence in setting agendas, convening stakeholders, and ensuring accountability. To nurture transformative local leaders for collaboration, holistic, equity-driven, community-informed approaches are essential. The study participants proposed several concrete steps: at the state level, establish “central management units” for supervising local implementers and ensuring bottom-up feedback; at the district level, rationalise committees and assign deliverables to stakeholders; and at the block level, expand convergence structures and involve local self-governments. Development partners can support data-driven priority setting, but local implementers with contextual familiarity should develop decentralised plans collaboratively, articulating rationales, activities, and resources. Finally, innovative training programs are required at all levels, fostering humility, motivation, equity awareness, leadership, problem- solving, and data use proficiency.

**Conclusion:**

This study offers multiple solutions to enhance local implementers’ engagement in multisectoral efforts, advocating for the development, piloting, and evaluation of innovative approaches such as the block convergence model, locally-led collaboration efforts, and novel training methods for local implementers.

**Supplementary Information:**

The online version contains supplementary material available at 10.1186/s12913-024-11002-2.

## Background

The importance of multisectoral collaboration for primary health care (PHC) is clear.

“*PHC is a whole-of-society approach to health that aims at ensuring the highest possible level of health and well-being and their equitable distribution by focusing on people’s needs, as early as possible along the continuum from health promotion and disease prevention to treatment, rehabilitation and palliative care, and as close as feasible to people’s everyday environment.*” [1].

The health sector alone cannot address the structural and social determinants of health necessary for PHC [2–5], and non-health sectors may play a greater role in improving population health outcomes. Addressing the determinants of health to improve health outcomes necessitates collaboration across sectors and community groups [3–5].

Such multisectoral approaches have been proven effective in improving health outcomes [6]. They have been prioritised in several global health commitments for PHC [7, 8] and recognised as key for nations to achieve the Sustainable Development Goals (SDGs) [9, 10]. An effective multisectoral approach addresses a problem from multiple dimensions, and encompasses various stakeholders, including government, civil society, the private sector, community structures, and individuals [11, 12].

The existing literature contains several useful insights on the nature of such multisectoral collaboration. Collaboration is a product of shared motivation, principled engagement, and capacity for joint action that is influenced by the overall systems context, and should be adapted based on emerging outcomes [13, 14]. Collaboration unfolds through stages, from simple sharing of information and resources all the way through to shared structures and merged remits across sectors [14, 15]. Such collaboration is not simple, but may need to occur across a number of processes and structures, from financial resources to reporting mechanisms [16].

In LMICs, multisectoral collaboration for health is commonly initiated for specific disease control objectives, primarily in response to disease outbreaks, but also in other priority programmatic areas such as Reproductive, Maternal, Newborn, Child and Adolescent Health (RMNCAH), formulating effective nutrition strategies, or managing non-communicable disease [5, 11].

There are many challenges in implementing multisectoral initiatives in LMICs. Institutional capacity can be low, and coordination can be compromised by fragmentation, even fragmentation within the health sector itself. Shifts in political commitment, leadership, and funding streams can also make maintaining such collaboration challenging. Other factors including inter-departmental hierarchies and weak organizational structures can also impede the process of collaboration. Working across sectors presents further challenges including persuading non-health stakeholders of the necessity for collaboration, creating governance and mechanisms for aligning diverse stakeholders’ interests, establishing priorities, and resolving differences toward a shared objective [11].

Given this context, Rasanathan and colleagues have outlined a roadmap to effectively govern multisectoral health actions in LMICs. Their proposed strategies include understanding key actors and the political ecosystem, framing the issue strategically, defining clear roles and interventions by sector, utilizing existing structures when feasible, addressing conflicts of interest, distributing leadership, establishing financing and monitoring systems, strengthening implementation processes and capacity, and fostering mutual learning and implementation research [17]. Other documented successes of multisectoral collaboration across a range of LMICs make a strong case to conceive multisectoral collaboration as an ongoing and iterative process, and highlight the need to support planning such dynamic approaches [18].

Nevertheless, previous reviews point out gaps in research, specifically in understanding the details of the implementation of multisectoral strategies. The literature does not contain robust identification and comparison of governance arrangements for multisectoral collaboration. There is also a lack of clarity regarding implementation processes, the role of contextual factors, and the capacity requirements for multisectoral collaborations [19]. Very few studies specifically describe tangible inputs required for implementers to execute multisectoral collaboration.

### Multisectoral collaborations efforts in India

Multisectoral collaboration forms an essential part of several health policy frameworks in India [20–24]. Acknowledging the importance of wider determinants, India’s National Health Mission (NHM) embraces a convergent approach to decentralised planning and action. Structures for multisectoral collaboration have been envisaged at the community level, in health facilities, and across different administrative levels from national to districts [20, 21].

At the village level, the Anganwadi Centres, childcare and nutrition centres under the Integrated Child Development Services (ICDS), are the primary hub for convergent health action. Comprehensive Primary Health Care (CPHC) is also delivered at the village level through a network of health centres. The Sub-Centre Health and Wellness Centres (HWCs) cater to 3000–5000 individuals at the grassroots level, while Primary Health Centres HWCs serve 20,000–30,000 in rural areas and up to 50,000 in urban areas. HWCs are expected to go beyond ambulatory primary health care and providing public health services. They have a mandate to nurture community participation and promote multisectoral collaboration to address social determinants of health [25, 26].

Anganwadi Centres, HWCs, and PHCs are directed to collaborate with local self-government structures called Panchayat Raj Institutions (PRIs) to achieve decentralization. The Village Health Sanitation and Nutrition Committees (VHSNCs) in every village are expected to pull together actors across sectors, including the health sector and PRIs, in order to carry out activities such as WASH and nutrition.

Above the health facility level, hospital management committees constituted from primary to secondary care health facilities are expected to foster multisectoral collaboration. The aim is to ensure quality healthcare through people’s participation, accountability, and transparent fund utilization.

Through coordination meetings, surveys, and feedback from the communities, these village and health facility based structures are expected to develop Village Health Plans feeding into District Plans. The idea is that collaborating across sectors and across levels will facilitate effective convergent action [20, 21, 25].

There also exist inter-departmental committees at higher levels on convergence for health. Inter-departmental coordination committees at the district level are the responsibility of district-level administrators called District Collectors, and are chaired by Mission Directors of NHM at the national and state level. District Collectors are administratively responsible for all sectors and have defined processes for collaboration. Also at the district level, the District Health Societies in NHM function as the central forum for stakeholders, including line departments, PRI, and civil society to engage in the planning, implementation, and monitoring of health and family welfare programs within the district [20, 27]. These decisions and actions are coordinated at the block (sub-district) level.

In the maternal and childhood nutrition and immunization programs in India, cross-sectoral collaboration has been observed in policy formulation, joint planning, reviews, and monitoring and evaluation [28, 29]. In these multisectoral collaboration efforts, leadership, including trust, motivation, and knowledge, is a key driver [15, 30]. Institutional factors matter too: these include the need for adequate skilled human resources, clear mandates, detailed planning, and well-coordinated relationships [29, 30].

Challenges in implementing multisectoral collaboration for health have been observed across various levels and initiatives in India. While a few studies on nutrition programs suggest that effective interpersonal communication among frontline providers leads to smooth collaboration at the grassroots level [15, 28], other convergence structures, such as the VHSNCs and hospital management committees, have faced limitations due to contextual barriers [31], insufficient financial management, and opaque governance processes. These obstacles have hampered organizational capacity for decentralization [32].

The sub-district and district levels, collectively referred to as the meso-level, are particularly important in primary health care related processes [33]. Earlier research conducted in India underscores numerous challenges faced by implementers at the meso-level, including insufficient supervision, a dearth of coordination tools, excessive workloads, and inadequate communication for multisectoral initiatives [15]. These levels also face challenges like inadequate capacity building, affecting fieldworkers, district managers, and health providers. Problems such as unclear role definitions, vertically structured supervision, and a lack of coordination in data monitoring add to implementation difficulties [28].

Although there is much insight on challenges, there is less in the literature regarding what might be done about them. The above insights on multisectoral collaboration at the meso-level in India, drawn from limited program-specific studies, are inadequate for prescribing precise approaches, processes, and capacity-building needs for improving meso-level stewardship in multisectoral collaboration for PHC. Similarly, evidence from other LMICs is also insufficient to provide recommendations to improve implementation and capacity requirements that can be applied to India [19].

A prior study in India highlights that creating institutional forums, and conducting joint consultations aligned with global and national policies, help in stakeholder engagement for multisectoral collaboration [34]. For certain programs, despite existing policies to promote stakeholder coordination, substantial opportunities remain to improve the joint planning, implementation, and monitoring of flagship programs [35]. However, the specific nature of what these improvements should look like at the meso-level remains unclear.

This paper aims to develop a more nuanced understanding of “How” to enable local implementers at the district and subdistrict level to advance, multisectoral collaboration for PHC. Our investigation also seeks to identify key issues faced by implementers in stakeholder engagement and elicit their recommendations for enhancing coordination and promoting effective multisectoral collaboration. We describe the common drivers, challenges, and - more importantly - working mechanisms with potential to enhance the implementation of multisectoral interventions, particularly at the district and subdistrict level. We also present insights on the perceived needs for capacity building and recommendations on training approaches to achieve impactful multisectoral collaboration by local health managers. We hope these findings will serve to strengthen multisectoral collaboration efforts at the district and sub-district levels in India.

## Methods

We used a purposive maximum variation sampling approach to select participants with significant experience of working on multisectoral initiatives for health. The research team members who have worked with India’s health systems identified information rich respondents and further requested them to suggest individuals who could provide insights for the implementation processes and strategies to enhance district and sub-district level multisectoral collaboration efforts for PHC. We conducted 20 semi-structured interviews, ensuring a heterogeneous group of interviewees to capture the widest range of perspectives possible, similarities and differences. The interviewees included national/state policymakers from health and allied departments, program officers/implementers at the state/district/block levels, representatives from development agencies, and academics from research and training institutions, all with experience in multisectoral initiatives. (See Table 1). To capture maximum variation in location, the diverse interviewee pool was obtained from ten different states in India-Bihar, Chhattisgarh, Delhi, Jharkhand, Karnataka, Kerala, Madhya Pradesh, Odisha, Tamil Nadu, Uttar Pradesh. These states were purposively identified based on research team members’ professional network and ability to recruit participants from these states. This facilitated examining multisectoral collaboration perspectives across different contexts, identifying significant common patterns that hold true across variations.

As most participants had experience of working on health-related intersectoral initiatives and were identified by research team members who have worked with India’s health systems, this sample size was sufficient to attain thematic saturation on dynamics that exist across India. There are of course many state-specific or other locally specific structures within India; this study was not designed to gain deep insights into specific local contexts. We focused on the common threads across a diversity of Indian contexts.

**Table 1:** Participant details

Category of study participants	Numbers interviewed
Policy makers	6
Implementers	7
Academics	4
Representative from development agencies	3
**Total**	**20**

**Fig. 1:**
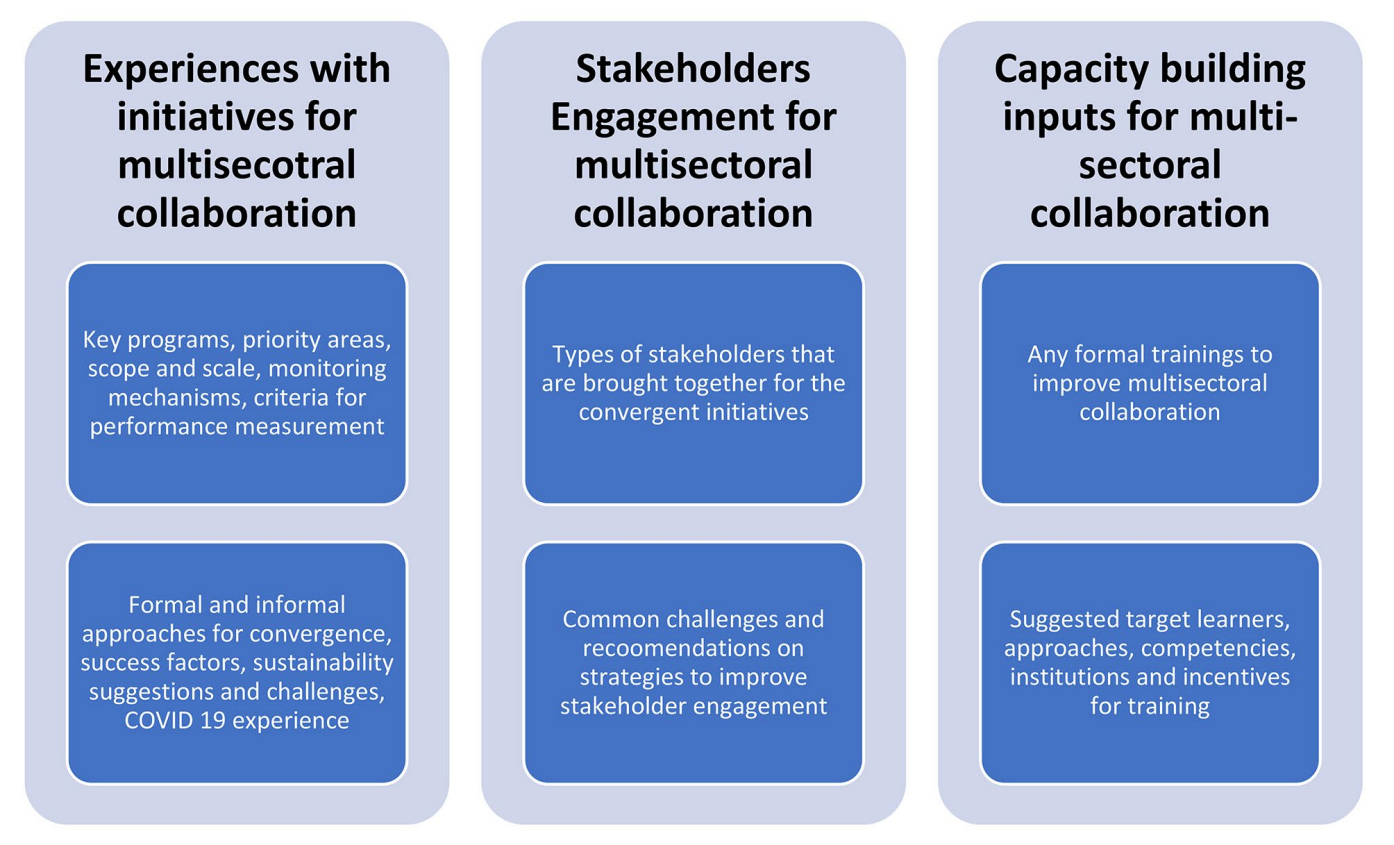
Research themes and domains covered

Our interview guide drew on Glandon et al.’s review of evidence on multisectoral convergence in LMICs [19]. The interviews primarily addressed participants’ experiences with multisectoral initiatives, the present status, facilitators, challenges, and their recommendations for enhancing stakeholder engagement and capacities, primarily focusing on the meso-level in multisectoral collaboration. The broad themes and specific domains covered in our interview guide are summarised in Fig. 1. Before data collection, the research team developed a series of a priori (deductive) codes based on these topical areas.

We conducted interviews remotely via Zoom and recorded them to the cloud through Zoom. Concurrently with data collection, we conducted multiple close readings of transcripts, prepared analytic memos, coded and finalised emerging themes. We conducted a rapid thematic analysis to synthesise prominent themes. Study results from the preliminary analysis were further fine-tuned with reflection upon the data and discussions among the research team members.

## Results

**Fig. 2:**
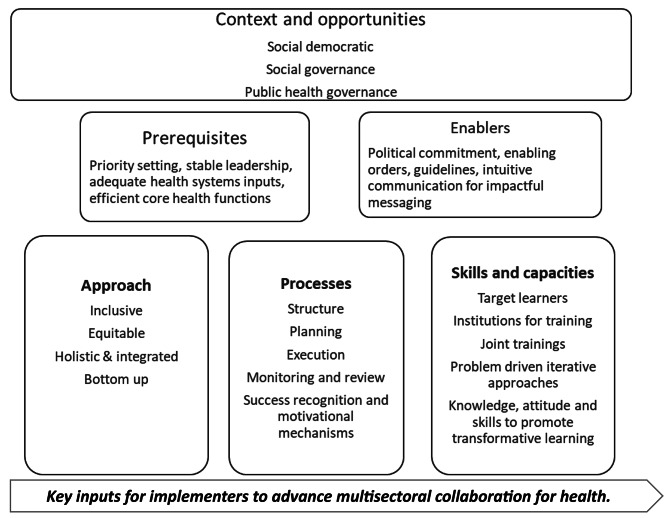
Explanatory framework for implementers to advance multisectoral collaboration for health in India

Our interaction with participants provided specific insights on multisectoral collaboration at district and sub-district levels in India. Participants emphasised the impact of context on how multisectoral efforts unfold and discussed “Prerequisites” as essential confidence-building conditions for implementers to get engaged in collaboration efforts. They identified “Enablers” as factors, tools, communication techniques enhancing collaboration. Their recommendation focused on three essential inputs to advance effective collaboration, particularly improving approaches, processes, and capacity building at the meso-level. “Approaches” pertain to the underlying ideas and mechanism for conceptualizing multisectoral action. Study participants referred to several “Processes” or a set of core activities for operationalizing multisectoral action. And “skills and capacities” construct dealt with the inputs for training required for multisectoral action. We organised these perspectives into an Explanatory framework for Implementers to enhance Multisectoral Collaboration for PHC in India (Fig. 2).

Here, we review each of these elements in turn.

### Context: creates opportunities and a culture of multisectoral collaboration

Deepening comprehension of the context surrounding multisectoral collaboration is crucial to grasp the factors that facilitate the success of or, conversely, hinder effective collaboration [36]. In our study successful multisectoral collaboration was mentioned to be facilitated by three distinct contexts: the social-democratic context in Kerala, the social governance context in Chhattisgarh, and the public health governance context in Tamil Nadu.

In Kerala’s social democratic context, the pursuit of development through social justice and democratic methods significantly influenced the shaping of multisectoral initiatives. Our participant from Kerala commented that the roots of their intersectoral action and community participation originated with two major equity-centred social movements: the pre-independence Sri Narayana movement, and the Shastra Sahitya Parishad of the 1960–1980s. These movements fostered a social justice orientation towards community problem solving and coupled with a democratic context through decades of local self-government empowerment efforts, allowed cross sectoral, community led approaches to become deep seeded in Kerala’s governance system. As an academic closely involved with implementation of Kerala’s primary health care reforms explained:

*Our experiments with the local governments… provided a basis for this multi sectoral approach towards addressing problems. And that sort of approach was sustained because the local governments had enough financial and political freedom to take decisions, implement the plan on their own and reap the benefits. (P-221122YR)*

He also mentioned how participation of local self-governments (Panchayat) was leveraged in planning Kerala’s flagship PHC program Mission Aardram.

*Before we implemented Mission Aardram we got the people from the local government, the president of the Panchayat Standing committee, a medical officer of the PHC, health instructors of the PHC, staff nurse of the PHC, a lab technician to understand the inputs and requirements of this new mission. Roughly 10 people, from a panchayat. Means right from the Panchayat President to the different functionaries they come together, we sit together. We discussed what are the problems, how do we approach them, what are the national programs that are available. What are the resources at your disposal? How can we comprehend the issues that we may have to address? (P-221122YR)*

In Chhattisgarh, a social governance context, characterised by people’s participation and ownership, in the government health programs has played a key role in building and maintaining structures for multisectoral collaboration. The VHSNCs created under the NHM serve as a platform for grassroots level convergence and are highly functional. An implementer from the state explained:

A *culture of social governance, where the government proactively engages and encourages people’s participation in decision making, played a key role in building effective convergence at the grassroots through the VHSNCs in our state*. *(P- 220922RG)*

In Tamil Nadu, a dedicated public health management cadre that ensured administrative leadership to be trained in public health and management competencies, enhanced health sector governance, fostering multi-sectoral initiatives. This system empowered public health officials to engage with other departments, enabling them to collect data, diagnose problems, identify convergence areas, and plan responsive actions. The cadre also played a crucial role in disease reporting and surveillance at primary health care facilities, by obtaining alerts from Panchayati Raj institutions. This ensures a continuous flow of health information to identify convergent action needs, particularly for waterborne illnesses and disease epidemics. A respondent perceived these mechanisms as effective in managing both routine health programs and the COVID-19 pandemic.

This policymaker from Tamil Nadu commented:

*An effectively managed public health cadre in Tamil Nadu provides robust leadership for convergence within health sector, supports the state and district officers of health with necessary authority to initiate communication related to multi-sectoral convergence. (P- 220901ZD)*

### Prerequisites and enablers for multisectoral collaboration

Pre-existing governance mechanisms lay the foundation for multisectoral collaboration in India. Other enablers are built on that bedrock. Adequate manpower, financing, and sound logistics systems maintain core health functions; guarantee operational efficiency within the health sector; and increase trust and confidence amongst the stakeholders to collaborate. Program officers said that their struggles to overcome these operational challenges impeded the quality and effectiveness of multi-sectoral collaboration and reduced the confidence of stakeholders from other departments in their capacities. A state health official observed that:

*We need to put our house in order first, address operational challenges, build internal collaboration, enabling mechanisms and systems for program officers to coordinate and converge within the health department …. this will also establish a culture and willingness to collaborate with stakeholders from other allied departments. (P-220914AA)*

A key prerequisite identified by our respondents was stable administrative leadership within the health sector. Stable tenures of health administrators allowed time for leaders to understand health sector functioning, identify challenges, and establish priorities for multisectoral collaboration.

In resource constrained settings of India, excessive workloads, competing priorities, and limited resources make it difficult to attract and sustain the interest of stakeholders from non-health departments towards collaborative action. One respondent from a state reflected on his experience of trying to explore willingness to work across sectors:

*I was quite shocked…to know there was not even one single person, one single secretary or officer in the government who said, I know what you’re saying is a good idea, maybe we should work on collaboration. …. Across it was a straightforward no….it was always, “No. Keep your health to yourself…. I’ve got a certain mandate I must do that, and if One Health helps means I have to change something and compromise on my department achievements, then I’m not ready to do it.” (P-221222NN)*.

In these situations, the success of convergent initiatives hinged on health leadership’s soft power and effective communication to make a compelling case for collaboration. A policymaker from Tamil Nadu emphasised that the effectiveness of such initiatives relies on implementers’ capacity for “intuitive communication,” that utilises instinct, understanding, or immediate comprehension. Without intuitive communication, engaging and maintaining the interest of stakeholders from non-health departments, especially those with competing priorities and limited resources, becomes challenging.

Participants emphasised the influential role of political leadership in successful multisectoral collaborations, citing examples such as India’s polio eradication program, Mission Indra Dhanush (MI) for childhood immunization, Japanese Encephalitis control in Uttar Pradesh, and the whole-of-government approach for managing the COVID-19 pandemic. The common thread in these initiatives was the active involvement of top political leaders at the national or state level, leading to accountability and regular monitoring of multisectoral efforts. Another common feature was bureaucratic leadership conducted joint data-driven reviews, identified gaps, and engaged in joint planning. What emerges is leadership played a vital role in enabling multisectoral action for various primary healthcare inputs- community mobilization, logistics, continuum of care, and governance.

For example, in the specific instances of Polio and MI program, political support facilitated collaboration between health and other departments like education, social welfare, rural development, etc., for effective community mobilization and vaccine delivery. In Uttar Pradesh, the Chief Minister led efforts to reduce Japanese Encephalitis deaths in seven districts, focusing on community awareness, sanitation, early detection, referral, and treatment. Here, the coordination mechanisms were established with municipal bodies for sanitation, for veterinary-based surveillance with animal husbandry department. In Odisha, dynamic leadership from the Chief Minister’s Office during the COVID-19 pandemic facilitated swift decisions and collaboration across departments for technical aspects such as drug procurement, infrastructure upgrades, bed availability, ambulances, and partnerships with private hospitals.

One state policymaker commented:

*For a program to succeed, somebody motivated must take ownership, whether it is at the state level or the district level. Somebody must be completely involved…. The higher the level of ownership, the wider the change that can be impacted. (P-220912IT)*

Proactive leadership further ensured that government orders and guidelines for multi-sectoral collaboration were shared across administrative levels and lead to action.

Beyond high-level leaders, local leaders matter enormously. Local leaders from Panchayat, tribal heads etc. are needed to bring health staff and communities together. One respondent commented that,

*“If systems want to reach marginalised and vulnerable communities, health systems representatives cannot do it on their own.” (P-220913AN)*.

### Equitable, empathetic and holistic approaches for multisectoral collaboration

Even with effective political leadership, though, our interviewees argued that there was a need for a shift in approach and values needed if multisectoral action for health was to be effective. Respondents pointed out that the framing of problems often had a restricted focus from the beginning, and was focused on coverage numbers, rather than the glaring inequalities and discrimination faced by women and children from vulnerable communities. *“There are very few multisectoral policies or interventions that directly address this challenge of inequalities and disparities*,” one respondent commented. Another academic observed:

*The issue is not about low immunization, per se, but about what low immunization coverage represents, which is that this community is vulnerable. Right now, our action is focused on increasing the immunization coverage, rather than reducing the vulnerability of a community overall.(P- 220913AN)*.

Our interviewees argued for different measures, aimed at evaluating the impact of a multisectoral initiative in terms of the extent to which it reduces inequalities and allows services to reach the hitherto excluded.

One policymaker shared their concern that in India’s hierarchial system, a lot of power is vested in civil servants who head districts, departments, or sectors. There is little scope for talking to people, understanding problems from people’s point of view, and developing solutions by engaging communities through a formal decision-making process. Our respondents argued that if the priority is to address social determinants, there is need for these approaches. Local leaders need to adopt empathetic, equity driven approaches. A state policymaker highlighted:

*What is lacking is a very interactive process of engaging with stakeholders, understanding what they want, giving local people a voice in decisions about their own development, about their own health care…. Responses to problems cannot be prescribed from the top…there is a need for understanding human problems…. humanizing the whole multisectoral collaboration and reaching people. (P-220913AN)*

Another problem is that district and block level action for multisectoral collaboration from health sector is usually led by doctors—who hold power at the local level. Our respondents commented that clinicians tend to over-prioritise clinical care:

*So, the biggest barrier is this understanding…you know the mindset… that the responsibility of health goes beyond the clinical care. (P-220922RG)*

Power relations were thus critical in effective multisectoral collaboration: if actors in power were empathetically oriented towards holistic approaches, multisectoral collaboration was much easier to carry out in practice.

### Process adaptations

Context, then, was very important in setting the stage for effective multisectoral collaboration. But within those broader contexts, respondents suggested a range of effective processes that could be implemented to support working across sectors to improve health. In these areas, they provided several actionable recommendations.

In addition to having the right mindset and ideology, participants discussed the critical importance of the right processes for multisectoral collaboration.

#### Structures of convergence

Inter-departmental task forces and committees form the governing structures of multisectoral action in India, at a variety of levels. Respondents articulated a need to rationalise their numbers, with better clarity on sectoral mandates and outputs. Some program implementers raised the concern that these platforms failed to enforce a notion of shared accountability. A district health officer commented:

*For me it’s a waste of time to participate in such committees…it’s multisectoral convergence for name’s sake. We do not contribute as there are no actions specified for the health department and no targets and outcomes are provided for tracking progress. (P-221009HA)*

Another implementer commented that there was not really accountability across sectors, but rather that the health department was still seen as the only accountable actor:

*So, if there are infant deaths…in general then they will hold the health department accountable, but not the Department of Women and Child Development. So, it gets structured that way. And that makes it very, very difficult to have effective convergence that sustains. (P-220922RG)*

The district and block implementers had several suggestions for making these structures more effective. One suggestion was to enable these committees to take on a more significant role at the state level, to serve as “the central management units.*”* While inter-departmental committees currently function to bring stakeholders together, enable policy formulation, and set priorities for multisectoral action at the state level, the state committees could potentially provide additional support for decentralised action by sharing uniform guidelines; identifying focal points at each district, block, and sub-block level for key decisions; establishing feedback systems like periodic progress reviews; and conducting troubleshooting exercises around the collaborative process.

#### Planning multisectoral action

The policymakers we interviewed recalled their experience of leading a multisectoral initiative and explained such planning in detail. “*Detailed action plans based on strong rationale and specifying the whys, how’s and whats are instrumental for successful multisectoral action,”* one respondent commented. He highlighted the key role of development partners in discerning the “whys for multisectoral convergence”. They can provide access to well analysed high-quality data and synthesise evidence for stakeholders to establish the rationale and priorities for convergent action. The implementation experience of the program officers in the field can determine “the how part” of multi-sectoral action. Stakeholders can jointly synthesise mechanisms through user friendly templates detailing key activities, roles, and responsibilities of key players in the health and allied sector, targets, timelines, and validation strategy for tracking outcome improvements. “The what’s of multisectoral action” are identified thorough elaborate resource mapping by each department.

Multisectoral collaboration is neither a one-time activity nor a periodic event, and implementing new programs requires a series of negotiations with different stakeholders. One policymaker explained that an implementation plan with clear articulation of needs for cooperation or support from other departments is extremely useful in approaching different stakeholders. Such planning can introduce efficiency in stakeholder engagement and save the time of already overburdened officers.

*So, then we need not involve all stakeholders together…. overall, it seems like a family is doing and involving itself in all stages, but every stakeholder need not sit together all the time… tracking the requirements… and we approach one department one at a time…. They will not even have time, and it is for us, then move systematically step wise. (P-220912IT)*

Another key informant mentioned that while template driven action planning is critical, it is important to provide flexibility at the district and block levels to allow responsive planning based on local context. The respondent mentioned a need to acknowledge issues each sector may face and suggested incorporating design thinking approaches in planning multisectoral action.

#### Execution at the district and block level

A need to strengthen district and block level implementation was pointed out by several participants. These was seen as the most complex levels, while the levels above (the state) and below (the village) seemed easier.

Respondents said that implementation suffers at the block level due to limited structures of convergence; the unfamiliarity of block representatives with collaborative problem solving; limited support for interpreting multisectoral guidance coming from the state; and human resource deficits. An experienced implementer explained that simply sharing guidelines is not sufficient:

*We assume that they will understand the program expectation from the orders which are released by the State government. But the challenge here is that they’re not able to figure it out. The perceptions of policymakers need to change… that sharing orders and leaving it for interpretation to the district/block teams may be sufficient…. So, training the stakeholders on joint orders and guidelines, their roles and responsibilities needs to be prioritised. (P-221124YJ)*

Block officials we interviewed called for expanding the scope of block intersectoral committees from being mere forums of review to participatory platforms for collaborative problem solving. One said:

*A review committee with inter-sectoral representation meets monthly under the leadership of a local self-government representative. Purpose of these meetings is however limited to reviewing financial expenditures and tracking progress of ongoing infrastructure projects in different sectors. It’s seldom focused on improving quality of care, population health or forging cooperation for joint problem solving. (P- 221124 S)*

To make progress at the block level, our respondents said, block models of broad-based multisectoral action are required. One implementer from Jharkhand mentioned:

*Yes…you keep coming up with some department specific projects, no problem…. But at least give one project to a block, which is a package of interventions focused on integrated development. And let the block team in that package …weave in an all-sectoral convergence…correctly spelling out who will do what. (P-221124YJ)*

Another expert involved in implementing one such block model of cross sectoral action for nutrition commented that such models are more effective and seamless if led by the Department of Rural Development and Panchayat Raj, the ministry responsible for local governance, rather than through health department structures. He explained:

*We implemented our integrated nutrition project involving multisectoral action with the Rural Development and Panchayat Raj (RDPR). As RDPR was implementing this model, it brought in these collaborations quite automatically……Unlike when health is the kind of the fulcrum, then the only party which is collaborating for nutrition programs is basically women and child department…. But when RDPR became the fulcrum, then the coordination became easier with … education, and water and sanitation etc. (P-221209HD)*

Increasing the role of Gram Panchayats (village level local governments) for multisectoral action emerged as a key strategy in several interviews. A participant from Karnataka explained how the Gram Panchayat Task Forces led the public health and humanitarian relief functions at the village level during the pandemic. The forums comprise of Panchayat electives, health providers, and members from vulnerable communities were trained and supported to perform complex tasks during the pandemic:

*Science based awareness was created by Gram Panchayats immediately by taking the information that we shared in our training based on the guidelines from the WHO and the Ministry of Health and Family welfare and translating them into local folk media… An effective COVID management was done by these forums. (P-220913AN)*

Participants from further Bihar and Jharkhand explained that Panchayat involvement can be planned as a long-term process, starting with orientation on their roles for health and enabling simpler levels of collaboration in terms of promoting health service access, leading later to more complex levels of engagement in planning, monitoring, and resource support for integrated development. Gram Panchayats can also lead community engagement for collaboration efforts by leveraging local women collectives (Self Help Groups) in social mapping and developing local action plans.

#### Monitoring and evaluation

Many respondents mentioned overcoming issues in data quality and efficiency as a critical input to improve monitoring of multisectoral initiatives. In some government departments, respondents articulated a need for more quality assurance in data gathering, and explained that there are often limited capacities, especially at the block level, to analyse the data and use that information to plan convergent action.

This issue is further complicated by the fact that there are multiple data sources used by different departments for measuring the same things in the same populations. A tendency to rely on data from one’s own department and blaming other data sources to be less accurate can lead to stakeholder clashes. As one respondent explained:

*Two different departments had different software… so there is a duplication of effort, the same cohort, the same parameters, but in two different software. Data being uploaded by two different people for the same village and the same individuals. And very often there are some discrepancies in the data… It also eventually leads to lack of ownership and stakeholder engagement issues. (P-221006AN)*

A distinct but related issue is that a lack of data and evaluation for multisectoral convergence, or even documentation of good models, can make advocacy for multisectoral collaboration challenging.

#### Motivational mechanisms

Good quality data is also necessary to map performance. Respondents argued for monitoring and rewarding good performance. One suggestion was to use simple mechanisms of performance monitoring based on district level data:

*You need to have disaggregated data, not the state level data, but district level data. So, you need to see what the district level data is that’s available on which you can base those incentives and you need to keep it simple… measure performance and you will also recognise performance. (P-220830JI)*

There are, of course, a wide variety of ways to reward good work. “*For some it’s monetary funding, for some it’s recognition, for some it’s pride,”* one respondent commented. What was shared between our respondents was the idea that recognition for good work was important.

One specific suggestion to increase collaboration between local self-government and the health sector was to introduce awards for the Gram Panchayats that have performed well on health-related indicators. A respondent explained that in the state of Kerala, Gram Panchayats improving public health activities and those contributing more funding for health are given financial awards by the government. This system could be replicated in other states.

### Building skills and capacity for multisectoral collaboration

Our participants unanimously agreed on the immense need for a training program exclusively devoted to multisectoral collaboration. One commented:

*So, multisectoral collaboration requires active facilitation. It does not happen on its own, and it does not happen just by providing it in the law or in the policy… mentioning it in the policy helps. Ah… but it also requires active facilitation…… it involves training. It involves supportive supervision. (P-220830JI)*

Interestingly, one policymaker mentioned:

*Management of COVID 19 pandemic and the whole of government approach deployed across all the states to overcome the health emergency has already provided two years of on-the-job training for enabling multi-sectoral convergence. Thus, willingness to converge for improving health outcomes is more now and needs to be continued and effectively leveraged*. *(P-221201AK)*

Respondents mentioned that such training programs should of course include learners from the health department, but also target learners from other allied departments, local self-government representatives, and bureaucrats.

Such training on multisectoral collaboration could take place both pre-service and in-service. One proposal was to integrate these trainings in the pre-service training programs for doctors and nurses, and into the Preventive and Social Medicine Curriculum for medical professionals. Another suggestion was to include these topics in the health module for civil servant trainees. And, one respondent commented, “*It would be essential to prioritise district and block program officers for any capacity building initiatives on multisectoral collaboration*.”

At the state level, administrative training institutes can serve as training sites for multi-departmental joint training on collaborative approaches. Training conducted under the leadership of the Kerala Institute of Local Administration (KILA) was cited as a potential model. There, newly elected self-government representatives were given governance training. This was a good place, one respondent commented, to “*call all these people together and conduct team trainings of all the sectors involved and the elected representatives.*”

Some respondents mentioned that moving beyond traditional didactic approaches to teaching might present an opportunity to refine and improve training outcomes. Several participants stressed context driven problem-solving approaches to training based on case studies, group problem solving and shared learnings. “*You should give them capsules of training,”* one respondent suggested, *“and that capsule of training should be based on what they are going to do in the field*.”

Another policymaker added:

*Regarding the approaches and strategy towards capacity building, innovative training methods such as Problem Driven Iterative Adaptation (PDIA), a new strategy for building capability by delivering results can be used…. A team of stakeholders from various departments of district can be invited for the training program. They identify a problem which they want to solve. Groups then follow a PDIA involving a step-by-step approach which helps them break down their problems into its root causes, identify entry points, search for possible solutions, plan convergent action, reflect upon what they have learned, adapt, and then act again.*

(P- 220901ZD)

There was also a felt need to link real world scenarios within training contexts. One suggestion was to bring together learners from districts with similar contexts, having common problems and challenges. The learners could be given an opportunity to identify root causes of poor health, identify entry points, search for possible solutions, and plan convergent action. They then could be supported post training to implement, reflect upon what they have learned, adapt, and then act again.

With reference to training content, participants mentioned understanding the country’s larger development agenda; explaining how multi-sectoral collaboration can contribute to the SDGs; to explain how improvements in one sector can yield improvements in outcomes for other sectors; to explore the concept of equity; and to develop skills for data gathering, analysis and identifying health disparities.

Skills for leadership, management, interdisciplinary teamwork were also identified as important. Since collaboration can seem burdensome or impractical to some, training programs could also include modules on communication and advocacy.

Respondents highlighted the need to convince both medical practitioners and civil servants of the value of multisectoral collaboration. “I think one of the critical aspects of multisectorality… is attitude,” one respondent commented. Another commented on the “attitude… especially among the health officials is a major source of stakeholder power dynamics. Doctors believe that we are the bosses, and if you want to collaborate, you collaborate as a silent spectator.” Without a shift in this attitude, another respondent argued that making progress on multisectoral collaboration could be difficult.

Here, training connects back to the enablers, positive stakeholder communication and political commitment necessary for multisectoral collaboration.

## Discussion

Multisectoral policies and actions are critical to strengthen primary health care. Our interviewees highlighted how context builds a governance environment, creates opportunities, and establishes foundational pre-requisites and enablers for implementers to forge convergence.

Not all models of context are replicable: the historical context of social governance found in Kerala or Chhattisgarh could be hard to create in other states. That said, there is scope to influence context. For example, the public health management cadre led governance model of Tamil Nadu may be replicated in other states. In particular, since a nationwide plan for a public health management cadre has just been created [37], there is a unique opportunity to increase capacities of local implementers and build administrative systems for inter-departmental partnerships across India.

Leadership and adequate health systems inputs as key determinants for collaboration corroborate with findings from other studies [11, 12, 38]. Here, power plays a significant role in influencing multisectoral endeavours, primarily through the leader’s ability to decide or establish the collaboration agenda [39].

We also know collaboration emerges from a shared motivation, principled engagement, the capacity to take joint actions, with sharing for information and resources throughout the collaboration journey [13, 14]. The recommendations from our participants on strengthening key inputs- approaches, processes, and capacity building for multisectoral collaboration address all these facets.

What specifically emerges in our study is the need to nurture transformative *local* leaders, who are willing to adopt holistic, equity driven, community informed approaches for responsive multisectoral action. Actors who hold power at the local level are key in promoting multisectoral collaboration. Such leaders can support careful thinking about collaboration with shared goals and responsibilities and help alleviate the power asymmetries, fragmented approaches, and prioritization of vertical programs that are common challenges observed while engaging development agencies [40].

Several process adaptations were highlighted by our participants that can improve sharing information and resources for multisectoral action. First, state level convergence structures serving as “central management units” with program officers and experts from different departments can help in supportive supervision of local implementers and ensure bottom-up feedback required for multisectoral problem solving. Also, rationalizing the number of district committees, as well as assigning deliverables and targets to stakeholders, can enhance the efficiency and accountability of multisectoral efforts.

Our study provides specific solutions to overcome the lack of performance of block-level implementors, highlighted as a challenge in other studies too [38]. Increasing block level structures of convergence, expanding their scope as working groups of joint planning and problem solving are useful suggestions. Also required are block models of broad-based multisectoral interventions, implemented with increased participation of local self-governments and steered by local rural development departments. The recently announced Aspirational Blocks Program of the Government of India provides an opportunity to pilot and evaluate such block convergence models.

The significant contribution of Gram Panchayats in the COVID-19 pandemic has been observed in different settings and led our respondents to confidently suggest a greater role of these organizations [41–43]. Gram Panchayats, with adequate training and support, are capable of handling complex health related activities and yield greater community collaborations when working with community-based collectives such as the Self-Help Groups [44]. However, given India’s diversity, our participants suggested a phased and a long-term plan for developing Panchayat-led models of convergence, starting with basic health promotion related activities to more evolved roles for them in planning and management of primary health care. Such steps would make sense in the context of India’s current policy emphasis towards greater ownership and additional financing to local self-governments for Primary Health Care [45].

Planning for multisectoral action will require bottom-up approaches. Our interviewees identified specific roles in planning for development partners and local implementers of different departments. Development partners can support data-driven priority setting to determine rationale for convergence that is responsive to evolving needs. Local implementers, with greater familiarity of context, sectoral strengths and weaknesses, should lead the activities and plan resources allocations for implementation. These two distinct roles allow each actor to leverage their respective expertise, promoting improved outcomes and mitigating the risk of defaulting to traditional power imbalances.

Evidence suggests “the skills required to support multisectoral action may be absent” [11, 46] as the public health workforce in LMICs receives limited training, restraining the effectiveness of the primary health care systems, and hence achievement of the SDGs [47].

Our respondents were unified in arguing that training is required to increase capacities for joint action, for health and non-health stakeholders. Leadership, management, communication, interdisciplinary teamwork, knowledge of SDGs and integrated development, the ability to recognise equity gaps, and competency in data use emerged as critical competencies that should be strengthened through training programs.

Other studies have also highlighted some of these capacity building inputs [48, 49]. Opportunities to optimise public health training in India suggest including better teaching-learning methods [50, 51]. Our study highlights that training techniques such as Problem Driven Iterative Adaptations (PDIA) can be effective learning methods for local implementers [52].

Throughout all these steps, attention to power is necessary. Making these intersectoral initiatives work would require “overcoming ego with humility” to reduce power dynamics and improve stakeholder engagement for intersectoral efforts [53]. Creative methods to target the attitude of civil servants and medical practitioners could allow for transformative learning for implementers.

### Limitations

This study provides multiple useful solutions to improve implementers engagement in multisectoral efforts. However, these perspectives are only from participants working in the government, and most of our respondents are working in health sector. Further research is needed to understand perceptions of private and non-health sectors on multisectoral collaboration. The external validity of these findings beyond the Indian context is uncertain. This study was a qualitative design, intended primarily for specific insights within India’s context rather than broad applicability. With the acknowledged importance of multisectoral collaboration in India, participants might have felt compelled to express favourable attitudes, potentially leading to social desirability bias. Despite these limitations, we maintain that the findings remain valuable and meaningful.

There is a strong case to develop, pilot and evaluate new approaches identified in this study to strengthen multisectoral convergence such as-the block level convergence model for integrated development, Gram Panchayats led collaboration initiatives and the new training methods suggested for local implementers such as the PDIA and mentored practicum techniques.

Finally, the existing multisectoral initiatives for health in India can be documented and evaluated for impact on health outcomes. Such studies would enrich the training programs and serve as an advocacy tool to encourage local implementers to collaborate.

## Conclusion

Our study highlights the need to be attentive to power; and the need to nurture transformative local leaders, who are willing to adopt holistic, equity driven, community informed approaches for responsive multisectoral action.

We identify several concrete steps towards this goal. At the state level, convergence structures serving as “central management units” can help in supportive supervision of local implementers and ensure bottom-up feedback. At the district level, our respondents recommended rationalizing the number of district committees, and assigning deliverables and targets to stakeholders. At the block level, key steps include increasing block level structures of convergence, and expanding the role of Gram Panchayats. While development partners can support data driven priority setting, local implementers with greater familiarity of context, sectoral strengths and weaknesses should lead the activities and plan resources allocations for implementation. Finally, training is needed to facilitate these processes at all levels.

### Electronic supplementary material

Below is the link to the electronic supplementary material.

Supplementary Material 1

## Data Availability

The datasets used and/or analysed during the current study are available from the corresponding author on reasonable request where participant privacy can be protected.

## Abbreviations

SDG: Sustainable Development Goals
LMICs: Low and middle-income countries
VHSNCs: Village Health Sanitation and Nutrition Committees
RDPR: Rural Development and Panchayat Raj
WHO: World Health Organization
KILA: Kerala Institute of Legislative Administration
PDIA: Problem Driven Iterative Approach
